# A Text Book of Physiology

**Published:** 1879-08

**Authors:** 


A Text Book of Physiology. By J. Fulton, M.B.C.S., Eng.; L. R. C. P. Lond, Prof. Phys. and Sanitary Science, Trinity Med. College Toronto, etc., etc. Second edition. Revised and enlarged, with numerous illustrations. Lindsay & Blakiston, Philadelphia. 1879. Price $4.00.

Through Messrs. J. J. & S. P. Richards, booksellers, we have been furnished the above work for review. Great progress has been made, of late years, in the science of physiology, and the microscope has been the means of discovering much that was unknown before its use. The author's conclusion, that histology is the key-stone to physiology is very correct, and the incorporation of the history of this branch of the study in the body of the work is a happy procedure.

In the first chapter on "proximate principles" the student will find greater ease in acquiring a knowledge of this fundamental part of the study, than in most other text books on physiology. In chapter second, third and fourth the origin, developement and forms of tissues, are discussed in full.

We commend the work to the student, and think it is the best text book on Physiology that has yet been issued.

---

Pamphlets Received: Complimentary Dinner given to Prof. S. D. Gross. By his medical friends, in commemoration of his fifty-first year in the profession, April 10, 1879. Lindsay & Blakiston, Phila. 1879.

The Treatment of Epithelioma of the Cervix Uteri. By J. Marion Sims, M.D., Founder of the Women's Hospital of N. Y., and formerly Surgeon to the same, etc., etc. Reprint from American Journal of Obstetrics and Diseases of Women and Children. Vol. xv., No. 111. July, 1879.

North Carolina Board of Health. Method for Performing Post-mortem Examinations. Prepared for the county superintendents.

Retroversion in Relation to Lacerations of the Cervix Uteri, etc. By Nathan Bozeman, M.D., New York.

National Board of Health Bulletin.

Transaction of the State Medical Society of Arkansas at its Fourth Annual Session. 1879.

The Demand for a Woman's Medical College in the West. By Chas. Warrington Earle, M.D., Prof. Diseases of Children. Chicago.

The Death Rate of St. Louis. An inquiry into the cause of its being less than of any other large city in the United States. By Chas. A. Todd, M.D., Prof. Physiology, etc., Missouri Medical College, St. Louis.

